# Soil fungal networks are more sensitive to grazing exclusion than bacterial networks

**DOI:** 10.7717/peerj.9986

**Published:** 2020-09-18

**Authors:** Lingling Chen, Jiajia Shi, Zhihua Bao, Taogetao Baoyin

**Affiliations:** Ministry of Education Key Laboratory of Ecology and Resource Use of the Mongolian Plateau & Inner Mongolia, Key Laboratory of Grassland Ecology, School of Ecology and Environment, Inner Mongolia University, Hohhot, China

**Keywords:** *Stipa glareosa*, Desert steppe, Grazing exclusion, Microbial communities, Co-occurrence networks

## Abstract

Soil microbial communities play a crucial role in ecological restoration, but it is unknown how co-occurrence networks within these communities respond to grazing exclusion. This lack of information was addressed by investigating the effects of eight years of grazing exclusion on microbial networks in an area of *Stipa glareosa* P. Smirn desert steppe in northern China. Here, we show that fungal networks were more sensitive to grazing exclusion than bacterial networks. Eight years of grazing exclusion decreased the soil fungal community stability via changes in plant composition and reductions in soil total organic carbon, in this case triggering negative effects on the *S. glareosa* desert steppe. The results provide new insights into the response mechanisms of soil microbes to grazing exclusion and offer possible solutions for management issues in the restoration of degraded desert steppe.

## Introduction

Grazing has a long history and remains the common land use type in Inner Mongolian grasslands (Bai et al., 2004). However, overgrazing has led to serious deterioration of grassland ecosystems in China during the past three decades (Hoffmann et al., 2008; Li et al., 2008). In response to regional environmental problems, the Chinese government launched some grassland restoration programs, such as the “Returning Grazing lands to Grasslands” in 2003 and the “Grassland Ecological Protection Program” in 2011, to mitigate grassland degradation by prohibiting grazing and increasing grassland vegetation biomass (Hao et al., 2014; Bryan et al., 2018). In these projects, various degraded grasslands were fenced for different numbers of years and grazing was permanently prohibited. Therefore, grazing exclusion has been widely used in Inner Mongolia for curbing grassland degradation and restoring damaged ecosystems (Bai et al., 2004; Hao et al., 2014). However, the effects of grazing exclusion are controversial depends on its vegetation types, environmental conditions, and duration (Jing et al., 2014; Yan & Lu, 2015; Yao et al., 2019).

Grazing exclusion can not only change ecosystem processes to promote the survival of local plants, but can also affect the activity and structure of soil microbial communities by increasing nutrient availability (Bastida et al., 2013; Zhang et al., 2018). The soil microbial community is a key driver of grassland ecosystems and of crucial importance for soil functioning (Bardgett, Wardle & Yeates, 1998). A major challenge is to understand how these complex communities respond to grazing exclusion. Many studies have demonstrated that grazing exclusion can have considerable effects on soil microbial communities, often with consequences for plant community dynamics (Cheng et al., 2016; Bi et al., 2018). It has also been shown that bacteria recover faster than fungi and with the rates being differentially governed by plant diversity (Zhang et al., 2018). Although these past studies provide important insights into grazing exclusion and microbial communities, up to now few data have been available concerning the impacts of grazing exclusion on soil microbial interactions.

Soil microbes can form a complex inter-species network that regulates the structure of ecological communities (Zhou et al., 2011; Lupatini et al., 2014). Evidence is mounting that the properties of these interaction networks can influence the response of soil microbial communities to environmental change (De Vries et al., 2018; Marcos, Bertiller & Olivera, 2019). In recent years, microbial network analysis has been used as a tool to explore the interactions in a range of environments, providing important details on microbial cooperation and competition (Faust & Raes, 2012; Yu et al., 2018). Additionally, microbial networks have been highlighted as crucial to understanding the dynamics of microbial community assembly and the responses of member interactions to a changed environment (Zhou et al., 2011). A previous study performed in arid region of Patagonia observed an increase in positive network connections, indicating that there are more cooperative relationships within the microbial community to survive under the stressful conditions imposed by grazing (Marcos, Bertiller & Olivera, 2019). In addition, a study performed in drylands of northern china showed that microbial interaction in extremely dry ecosystems is more sensitive to changes in water availability (Wang et al., 2018). Although the use of network analysis has increased in drylands, little has been revealed on how soil microbial interactions respond to grazing exclusion, especially in desert grassland ecosystems.

The desert steppe of Inner Mongolia cover about 20% of China’s desertification land area, which is characterized by harsh physical environments, infertile soil, and simple plant community composition (Wang et al., 2020). These natural ecosystems support the life of about 95% of the human population (Li et al., 2008). However, drought and dust storms have become the largest meteorological disasters in this area causing sparse vegetation and land desertification, which are exacerbated by overgrazing (Wang et al., 2020). In this study, we designed to assess the effects of eight-years of grazing exclusion on soil bacterial and fungal networks. We also aimed to test the changes in plant and soil properties and investigated how these changes affected the response of bacterial and fungal networks to grazing exclusion.

## Materials and Methods

### Study site

The study site was located in Erlianhot (43°38′34.44″−43°38′54.29″N, 112°07′58″E, 952–955 m elevation) of Inner Mongolia, northern China. The area has an arid and semiarid climate and is highly prone to drought, receives little precipitation, and frequently has strong winds. The annual mean temperature is 3.4 °C, annual mean precipitation is 142.2 mm, and approximately 70% of the annual rainfall occurs from July to August. The main soil types in this area are Aeolian sandy soils, with low fertility and loose structure. The representative vegetation includes *S*. *glareosa*, *Cleistogenes songorica* (Roshev.) Ohwi, and *Asparagus cochinchinensis* (Lour.) Merr communities, but the vegetation has been greatly altered by long-term overgrazing.

### Experimental design and sampling

The experiment was conducted in late August 2017, when grassland biomass was at its peak. We selected one site with two treatments: grazing excluded (GE) and grazed (G). Inside the grazing excluded area (comprising an area of 200 m × 200 m paddock), livestock has been excluded from this region since 2009 and the remaining site in the area was subjected to free grazing by sheep continuously all the year. Within the grazed and grazing exclude area, we established three 50 × 50 m (50 m apart) plots, respectively (Fig. S1). At each of the three plots, three 1 m × 1 m quadrats were randomly selected to investigate vegetation height, species number, and aboveground biomass. The number of species was used to estimate the richness of the plant communities, and the diversity of the vegetation was calculated using the Shannon–Wiener index (*H*′ = − ∑*Pi* ln*Pi*, where *Pi* is the ratio of the number of each species to the total number of all species). Plant aboveground biomass was harvested by clipping standing plant species to 1 cm above the ground. Plant material was then oven-dried at 65 °C for 48 h. To assess the effect of grazing exclusion on plant community structure, all plant species were classified into six functional groups: annuals and biennials (AB), perennial rhizome grasses (RG), tall perennial bunchgrasses (TG), short perennial bunchgrasses (SG), shrubs and semishrubs (SS), and forbs (FB).

Soil samples were collected from the top 20 cm of the soil profile using an auger after the vegetation was sampled. Nine soil cores were collected from each plot (three core points for each quadrat) and then mixed into a single soil sample. A total of six soil samples were collected from two study areas and sieved immediately and air-dried for physicochemical analyses. Next, ten plants of *S*. *glareosa* per plot were excavated carefully with adhering rhizosphere and bulk soil. The soil that could be shaken off easily from the roots was defined as bulk soil, and the soil that remained firmly adhered to the root was defined as the rhizosphere. At each of the three plots, the rhizosphere and the bulk soil samples of the ten plants were mixed into a single soil sample separately. The soils were sealed in 15 ml sterile plastic tubes, immediately frozen on dry ice, and stored at −80 °C until DNA extraction.

### Soil physicochemical analyses

The soil samples were sieved through a 2-mm mesh to remove roots and then ground and homogenized with a mill (MM400, Retsch, Germany). 15 g of fresh soil was weighed before and after dried at 105 °C for 24 h to calculate the soil water content (SWC). The soils were subjected to Kjeldahl digestion, and the soil total nitrogen (TN) concentration was determined using a semi-autoanalyzer (Kjeltec 2300 Analyzer Unit, Foss Tecator, Sweden). Soil total phosphorus (TP) concentration was measured by persulfate oxidation followed by colorimetric analysis (Schade et al., 2003). Soil total organic carbon (TOC) was determined with a TOC-5000A analyzer (Shimadzu Corp., Kyoto, Japan). Soil pH was measured using a ratio of soil:water = 1g:2.5 ml with a Delta pH-meter (Mettler-Toledo Instruments, Columbus, OH, USA).

### DNA extraction and high-throughput sequencing

Three replicate rhizosphere and bulk soil samples were used for DNA extraction. DNA was extracted from 0.5 g of soil with a FastDNA^®^ SPIN Kit for soil (MP Biomedicals, Santa Ana, CA, USA) according to the manufacturer’s instructions. The final DNA concentrations and their purity levels were determined using a NanoDrop^®^ ND-1000 (Nanodrop, USA), with DNA quality checked by 1% agarose gel electrophoresis. The DNA was then stored at −80 °C until use. The V3–V4 hypervariable region of the bacterial 16S rRNA gene was amplified using the specific primers 341F (5′-CCTAYGGGRBGCASCAG-3′) and 806R (5′-GGACTACNNGGGTATCTAAT-3′) *(Mori et al., 2014)*, and the fungal internal transcribed spacer 1 (ITS1)-ITS2 region was amplified using the primers ITS1F (5′-CTTGGTCATTTA GAGGAAGTAA-3′) and ITS2R (5′-GCTGCGTTCTTCATCGATGC-3′) (Bokulich & Mills, 2013; Hugerth et al., 2014). All PCR reactions were conducted with Phusion^®^ High-Fidelity PCR Master Mix (New England Biolabs). The amplifications were conducted in a 30 µl mixture including 15 µl of Phusion High-Fidelity PCR Master Mix (2 ×), 0.2 µM forward and reverse primers, 10 ng of template DNA, and PCR-grade water up to 30 µl. The amplification program was 98 °C for 1 min and 30 cycles of 98 °C for 10 s, 50 °C for 30 s, 72 °C for 30 s, and 72 °C for 5 min. After PCR amplification, the obtained products were purified using the GeneJET Gel Extraction Kit (Thermo Scientific Inc., USA) and subjected to quantification using a Qubit^®^ 2.0 Fluorometer (Thermo Scientific Inc., USA) and an Agilent Bioanalyzer 2100 (Agilent Technologies, USA) system. Last, the library was sequenced on an Ion S5™ XL (Thermo Fisher Scientific Inc., USA) platform and 600 bp single-end reads were generated at the Novogene Bioinformatics Technology Co., Ltd., Beijing, China. The bacterial and fungal raw sequence data from this study were deposited in the NCBI Sequence Read Archive (accession number PRJNA627740).

### Bioinformatics and diversity analyses

Single-end reads were assigned to samples using Cutadapt version 1.9.1 (Martin, 2011). The reads were compared with the reference database (Gold database) using the UCHIME algorithm to detect chimera sequences (Edgar et al., 2011), and then, the chimera sequences were removed (Haas et al., 2011). Effective tags were ultimately obtained. The sequence analysis was performed using Uparse software (version 7.0.1001) (Edgar, 2013), and the sequences with ≥97% similarity were assigned to the same operational taxonomic unit (OTU). The most abundant sequence in each OTU was chosen as the representative sequence. The bacteria were identified using the Silva Database (https://www.arb-silva.de/) (Quast et al., 2013) based on the RDP classifier (version 2.2) (Wang et al., 2007). The fungi were identified using the Unite Database (https://unite.ut.ee/) based on the Blast algorithm in QIIME software (version 1.9.1) (Koljalg et al., 2013). The phylogenetic relationships of the OTUs and the differences among dominant species in samples (groups) were analyzed using multiple sequence alignments in MUSCLE software (version 3.8.31) *(Edgar, 2004)*. The bacterial and fungal communities were characterized in terms of diversity by calculating Shannon-Wiener index and number of OTUs (richness) using QIIME software (version 1.9.1) (Caporaso et al., 2010).

### Network analysis

To make the bacteria and fungi networks comparable, the relative abundance of OTUs was log_10_-transformed and analyzed using the molecular ecological network analyses (MENA) pipeline (http://ieg4.rccc.ou.edu/mena) implemented with random matrix theory (RMT)-based algorithms (Deng et al., 2012) and then visualized with Cytoscape 3.7.1 (*Shannon et al., 2003*). The thresholds in the network construction were automatically chosen, and module separation was based on the fast greedy modularity optimization (Zhou et al., 2010; Deng et al., 2012). Modularity (*M*) is an index measuring the extent to which a network is divided into modules, and *M* > 0.4 was used as the threshold to define modular structures (Newman, 2006). Keystone species (module hubs and connectors) were identified by values of within-module connectivity (*Zi*) and among-module connectivity (*Pi*), where module hubs (highly connected to many nodes within modules) have *Zi* > 2.5 and *Pi* ≤ 0.62 and connectors (highly linked to several modules) have *Zi* ≤ 2.5 and *Pi* > 0.62) (Zhou et al., 2011).

### Statistical analysis

One-way ANOVA was used to evaluate the effects of the grazing exclusion on plants (richness, diversity, aboveground biomass), soil properties (TN, TP, SOM, and pH), and soil microbes (observed OTU richness and Shannon-Wiener index), followed by Tukey’s HSD test. These statistical analyses were conducted using SPSS 21.0 (Armonk, NY, USA). The changes in each variable induced by grazing exclusion were indicated as the natural log-transformed response ratio, *lnR* = *ln*(${\overline{X}}_{\mathrm{E}}$) −*ln*(${\overline{X}}_{\mathrm{C}}$), where ${\overline{X}}_{\mathrm{E}}$ and ${\overline{X}}_{\mathrm{C}}$ are the mean values of the variables that were observed in experimental group and control group, respectively (Hedges, Gurevitch & Curtis, 1999). The variance of *lnR* was calculated using the following equation: $\nu = \frac{({\mathrm{SD}}_{\mathrm{E}})^{2}}{{n}_{\mathrm{E}}{\overline{X}}_{\mathrm{E}}^{2}} + \frac{({\mathrm{SD}}_{\mathrm{C}})^{2}}{{n}_{\mathrm{C}}{\overline{X}}_{\mathrm{C}}^{2}} $, where SD_E_ and SD_C_ are the standard deviation for the experimental group and control group, respectively; *n*_E_ and *n*_C_ are the sample size (number of replicates) for the experimental group and control group, respectively. Principal component analysis (PCA) at OTU level was used to visualize patterns in bacterial and fungal community structures. Differences in bacterial or fungal community composition between grazed and grazing-excluded grasslands were tested using analysis of similarity (ANOSIM) tests with Bray–Curtis in the ‘vegan’ package, in R software (v2.15.3). Pearson correlation was used to analyze the effect of grazing exclusion on the relationship between the plant characteristics, soil properties, and soil microbial diversity.

Random networks with the same number of nodes and links were constructed to compare with the original network to determine general network characteristics (Zhou et al., 2011). For each identified network, 100 randomly rewired networks were generated, and all network indexes were calculated individually (Maslov & Sneppen, 2002). The *Z*-test was used to test index differences between the molecular ecological networks and random networks, and the Student *t*-test was used to test for differences in network indexes under different conditions using standard deviations derived from the corresponding random networks. The relationships between molecular ecological networks and environmental factors (plant characteristics and soil properties) were analyzed with Module-EigenGene analyses (Zhou et al., 2011; Deng et al., 2012). Spearman correlations coefficients were calculated to depict the relationship between the abundance of microbes and environmental factors. Results with a *P* < 0.05 were considered statistically significant.

**Figure 1:**
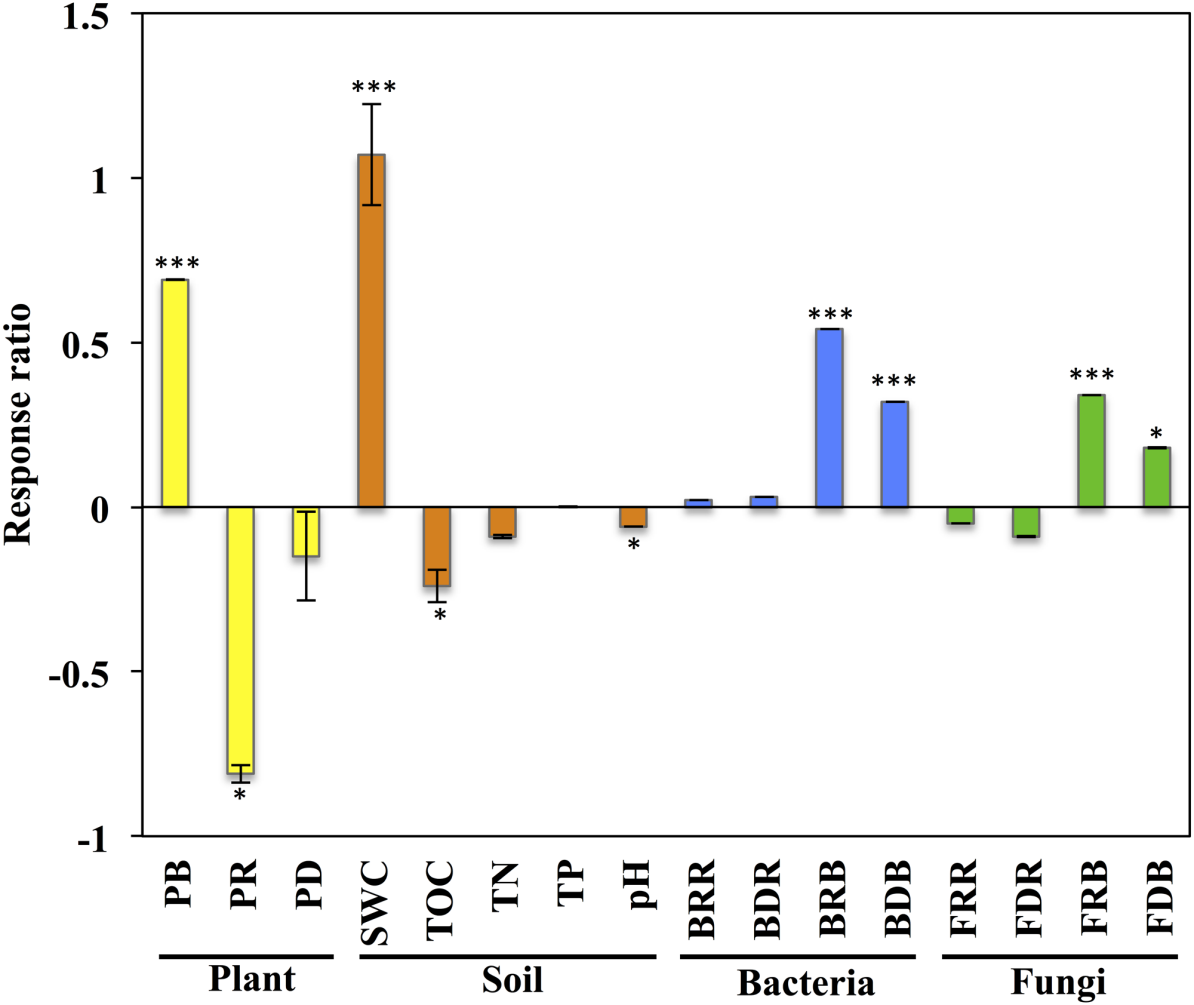
Response of variables of plants, soil, and soil microbial communities to grazing exclusion on the *Stipa glareosa* desert steppe of Inner Mongolia. An effect size >0 indicates a positive response to grazing exclusion, and an effect size >0 indicates a negative response to grazing exclusion. Data means ± SE (*n* = 3). The level of significance is as follows: ^∗∗∗^*P* < 0.0001, ^∗^*P* < 0.05. PB, plant aboveground biomass; PR: plant species richness; PD: plant diversity; SWC, soil water content; TOC: soil total organic carbon; TN, soil total nitrogen; TP: soil total phosphorus; pH, soil pH value; BRR: bacterial richness in rhizosphere; BDR, bacterial diversity in rhizosphere; BRB, bacterial richness in bulk soil; BDB, bacterial diversity in bulk soil; FRR, fungal richness in rhizosphere; FDR, fungal diversity in rhizosphere; FRB, fungal richness in bulk soil; FDB, fungal diversity in bulk soil.

## Results

### Responses of plant characteristics and soil physicochemical properties

**Figure 2:**
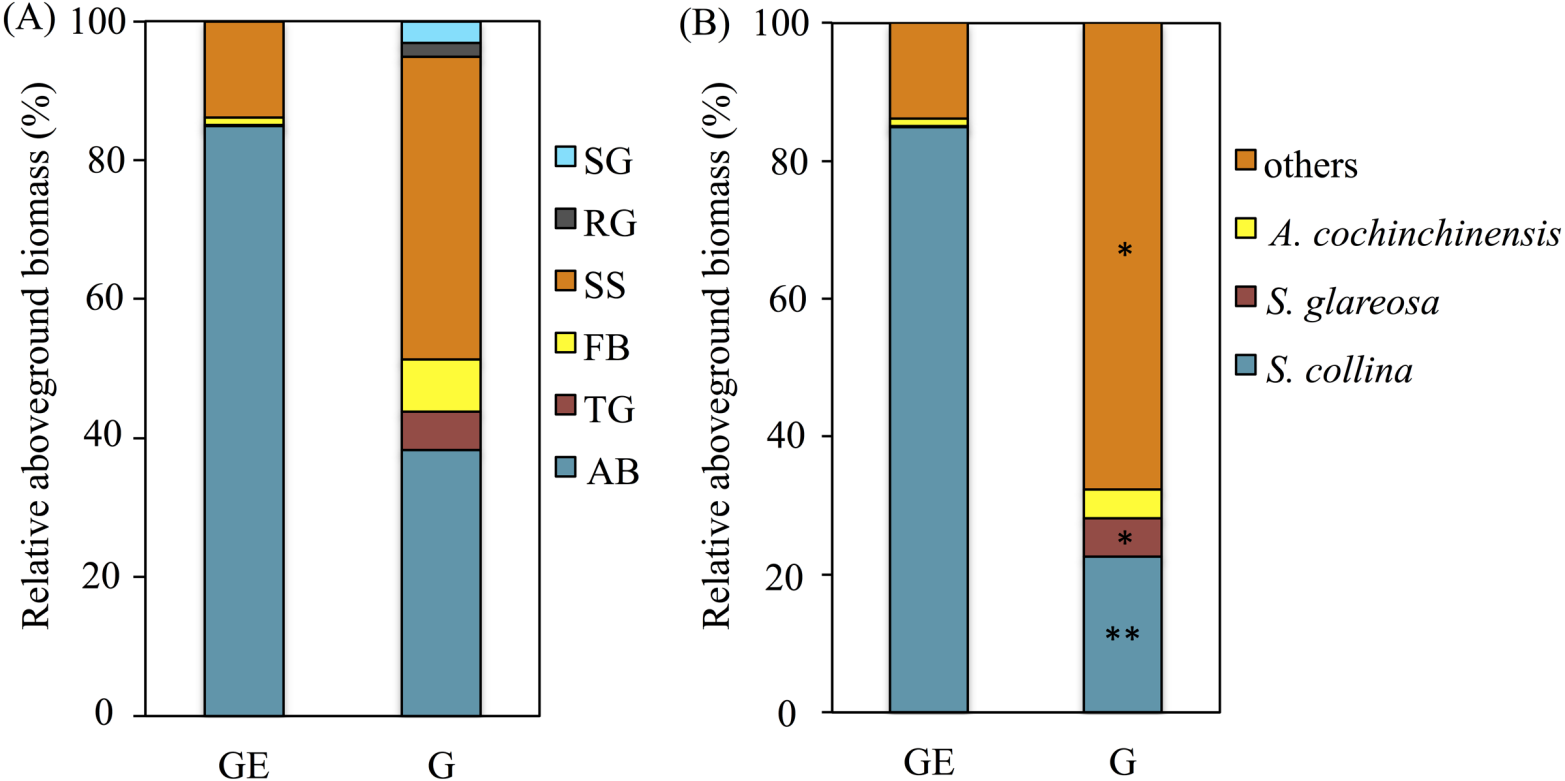
Effects of the grazing exclusion on relative aboveground biomass of plant functional groups (A) and dominant plant species (B) in *Stipa glareosa* desert steppe of Inner Mongolia. AB, annuals or biennials; FB, forbs; RG, perennial rhizome grasses; SG, perennial short bunchgrasses; SS, shrubs or semi-shrubs; TG, perennial tall bunchgrasses. G, Grazed area; GE, Grazing-excluded area.

The plant aboveground biomass had significant positive responses to eight years of grazing exclusion (*P* < 0.001), but the response of plant species richness was instead negative (*P* < 0.05; Fig. 1). For soil properties, SWC responded in a positive and significant way to eight years of grazing exclusion (*P* < 0.001), whereas TOC and soil pH had negative, significant responses to eight years of grazing exclusion (*P* < 0.05). The relative aboveground biomass of AB (dominant species *Salsola collina* Pall.) dominated in the grazing excluded grassland (Fig. 2). By contrast, the TG (dominant species *S. glareosa*), RG (dominant species *Leymus chinensis* (Trin.) Tzvel), SG (dominant species *C. songorica*), SS (dominant species *Caragana stenophylla* Pojark), and FB (dominant species *A*. *cochinchinensis*) decreased after eight years of grazing exclusion.

### Responses of soil bacterial and fungal communities

**Figure 3:**
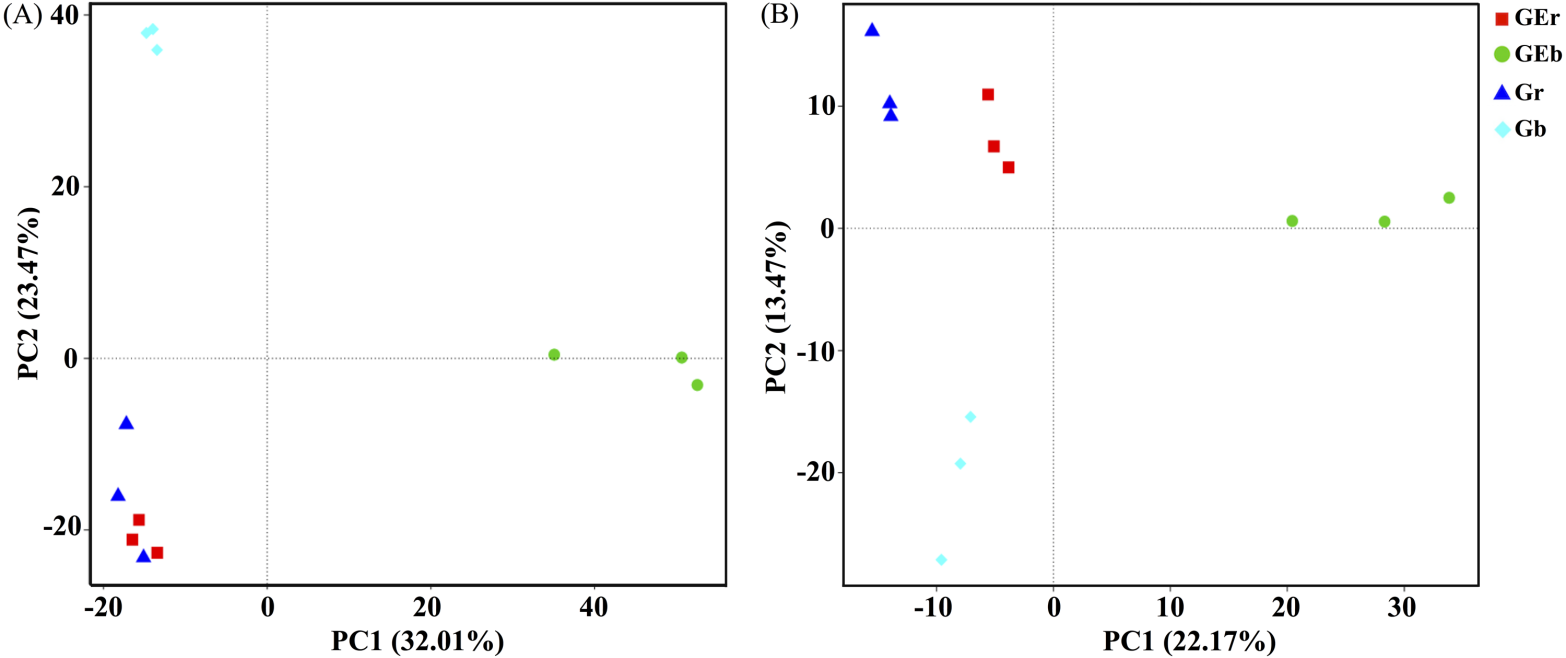
Principal component analysis (PCA) of bacterial (A) and fungal (B) community structures found at the grazed and grazing excluded grassland soils. GEr, rhizosphere soil of grazing-excluded plot; GEb, bulk soil of grazing-excluded plot; Gr, rhizosphere soil of grazed plot; and, Gd, bulk soil of grazed plots.

After quality filtering, a total of 953,050 bacterial sequences and 966,095 fungal sequences were obtained in this study, and clustered into 2,270 and 1,232 OTUs for bacterial and fungal communities, respectively. Eight years of grazing exclusion did not affect the richness and diversity of bacteria and fungi in the rhizosphere, but significantly increased (*P* < 0.05) them in the bulk soil (Fig. 1). The communities of bacteria and fungi were clearly separated between the grazed and grazing-excluded grassland soils (Fig. 3). However, no significant (*P* > 0.05) differences were detected in bacterial and fungal community composition between grazed and grazing excluded grassland soils (Table S1).

Among the highly abundant phyla, *Proteobacteria* had the highest abundance in the rhizosphere (38.96%) and bulk soil (34.94%) of grazing excluded grassland soils, followed by the *Actinobacteria* (29.38% and 31.02%, respectively), but their abundance were significantly decreased in bulk soil of grazed grassland (Fig. S2A). *Firmucutes* were the most abundant in the bulk soil (58.32%) of grazed grassland, but significantly decreased in grazing excluded grassland soils. We also observed two fungal phyla dominated the soil microbes: *Ascomycota* and *Basidiomycota* (Fig. S2B). In grazing excluded grassland soils, the *Ascomycota* had the highest abundance in the rhizosphere (58.03%) and bulk soil (83.08%), followed by the *Basidiomycota* (41.34% and 9.98%, respectively) and significantly higher in the rhizosphere. In grazed grassland soils, *Ascomycota* had the highest relative abundance in the rhizosphere (84.25%) and bulk soil (79.32%), followed by the *Basidiomycota* (9.98% and 1.37%, respectively).

### Response of soil microbial networks

**Figure 4:**
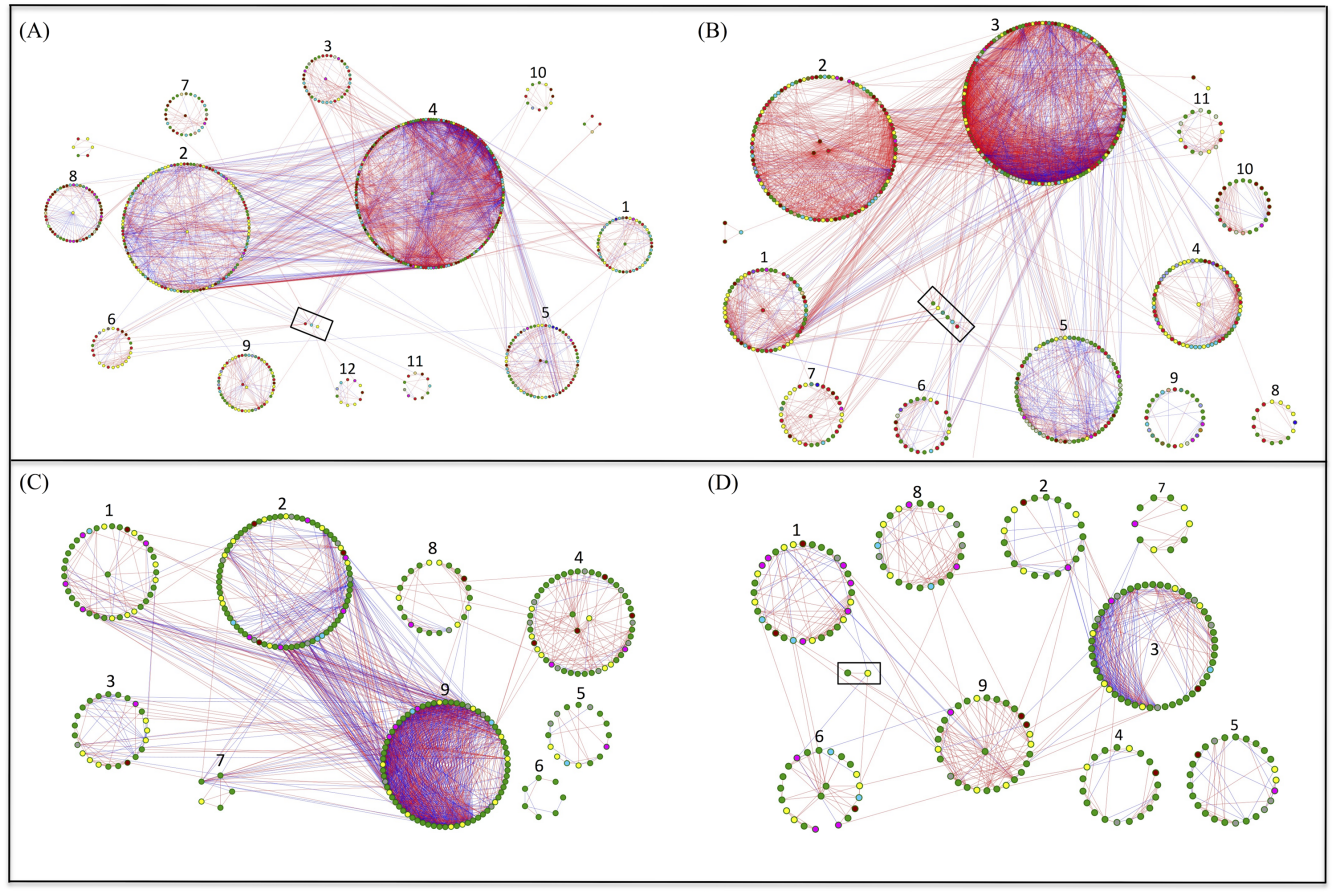
Highly connected modules of bacteria and fungi in grazed and grazing-excluded grassland soils. A red link indicates a positive correlation between two individual nodes, whereas a blue link indicates a negative correlation. Nodes at module centers are module hubs, and nodes in black boxes are connectors. (A) Bacterial network of grazing-excluded grassland soil; (B) bacterial network of grazed grassland soil; (C) fungal network of grazing-excluded grassland soil; (D) fungal network of grazed grassland soil.

Network analysis for bacteria and fungi in the grazed and grazing-excluded grassland soils, including data of both rhizosphere and bulk soil (since a significant rhizosphere effect was not detected), revealed that the networks of bacteria and fungi in the grazing-excluded grassland soil were larger than those in grazed grassland soils (Fig. 4, Table S2). However, grazing exclusion slightly decreased the proportion of positive links in bacterial networks from 76.58 to 68.32% and substantially decreased them in fungal networks from 69.33 to 57.50% (Table S2). The fungal network in the grazing-excluded grassland soils was more densely connected than those in grazed grassland soils, with more links, higher average degree and shorter path distance (Fig. 4, Table S2). Opposite trends were observed for the bacterial network in the grazing-excluded grassland soils. The value of *R* ^2^ of the power law ranged from 0.71 to 0.85. The values of the average clustering coefficient (avgCC), path distance (GD), and harmonic geodesic distance (HD) of all networks were larger than those for randomized networks (Table S2).

**Figure 5:**
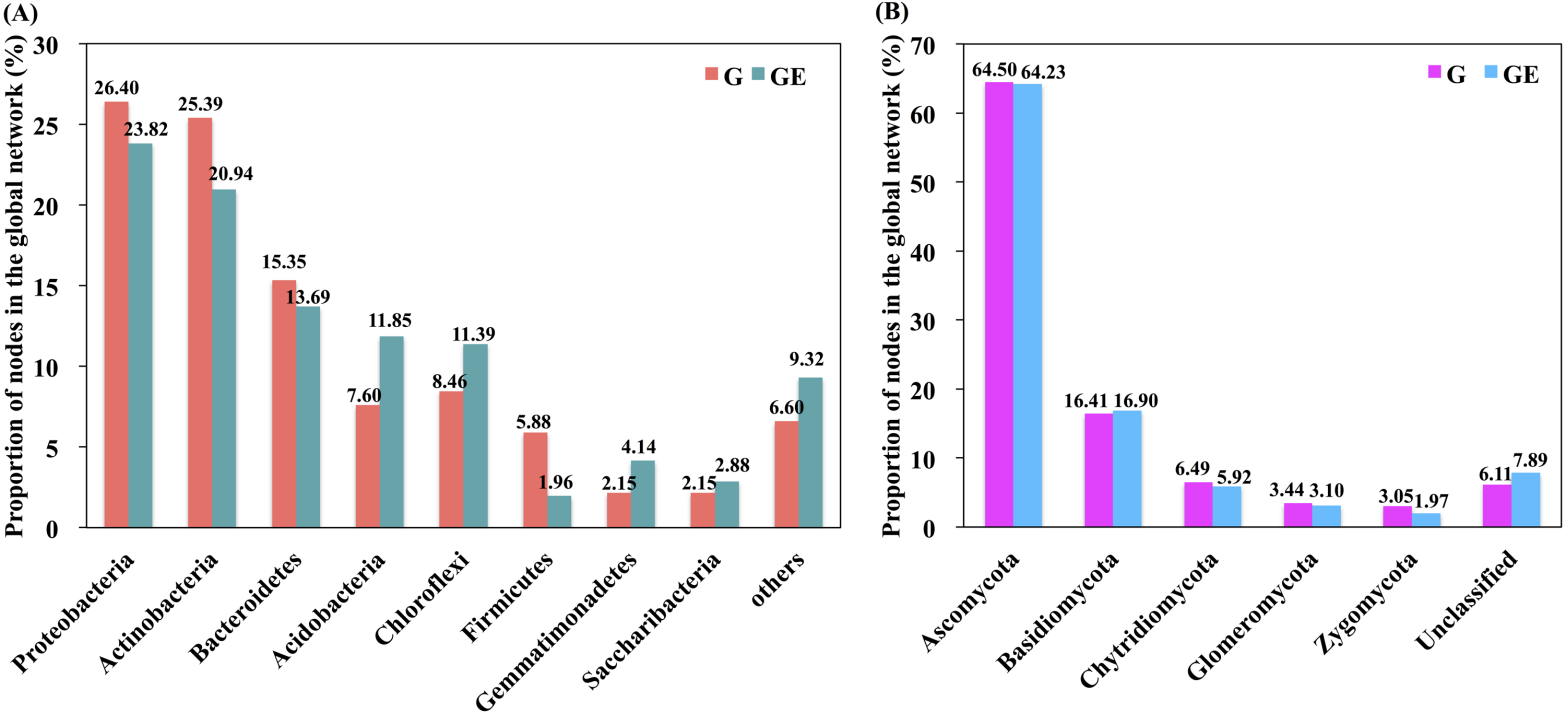
Proportion of bacterial and fungal nodes in global networks based on the eight most abundant bacterial phyla (A) and five most abundant fungal phyla (B). G, Grazed area; GE, Grazing-excluded area.

A total of six module hubs and six connectors were identified in the bacterial networks of grazed grassland soils, whereas 12 module hubs and three connectors were found in the bacterial networks of grazing-excluded grassland soils (Table S3). For fungi, we recorded three module hubs and two connectors in the grazed grassland soils, whereas, four module hubs were recorded in grazing excluded grassland soils (Table S4). In addition, approximately 80% of the bacterial nodes of global networks belonged to the phyla Proteobacteria (23.82∼26.40%), Actinobacteria (20.94∼25.39%), Bacteroidetes (13.69∼15.35%), Acidobacteria (7.60∼11.85%), and Chloroflexi (8.46∼11.39%) (Fig. 5A). The grazing exclusion network had higher proportions of Acidobacteria, Chloroflexi, Gemmatimonadetes, and Saccharibacteria compared with the grazing network. For fungi, the majority of the fungal nodes belonged to Ascomycota (64.23∼64.50%) and Basidiomycota (16.41∼16.90%) (Fig. 5B). The proportion of major fungal nodes in the global networks did not differ between grazed and grazing-excluded grassland soils at the phylum level.

### The correlations between plants, soil variables, and soil microbial community

Most variables examined in this study were correlated with one another (Table S5). Richness and diversity of bulk soil bacteria were positively correlated with (*P* < 0.05) plant diversity, TOC, and soil pH (Table S5), while that of bulk soil fungi were positively correlated (*P* < 0.05) with plant aboveground biomass and SWC, but negatively correlated (*P* < 0.01) with soil pH. The richness and diversity of the rhizosphere fungi was positively correlated (*P* < 0.05) with TN and soil pH (Table S5).

Eigengen analysis was used to reveal the relationships between network structure and environmental factors based on Spearman’s correlation analysis (Fig. S3). The bacterial network grouped to 17 and 11 submodules in the grazing-excluded grassland soil and grazed grassland soil, respectively, whereas the fungal network grouped to 9 submodules in both grazing-excluded and grazed grassland soils. The bacterial network of the grazing-excluded grassland soil was strongly correlated with plant diversity (Fig. S3A), whereas the fungal network was strongly correlated with soil TOC, TN, TP, and pH (Fig. S3C). In the bacterial network, the module 10 of grazing-excluded grassland soil was positively correlated with plant diversity and aboveground biomass (*P* < 0.05; Fig. S3A), whereas the module 9 of grazed grassland soil was positively correlated with plant aboveground biomass (*P* < 0.01; Fig. S3B). In the fungal network of grazing-excluded grassland soil, module 6 was positively correlated with soil TP content (*P* < 0.05; Fig. S3C).

## Discussion

### Effects of grazing exclusion on plant characteristics and soil physicochemical properties

The results of our study demonstrated that eight years of grazing exclusion on the *S. glareosa* desert steppe had negative influences on grassland ecosystems, because the primary plant communities (*S. glareosa*) were replaced by secondary plant communities dominated by *S. collina*, leading to retrogressive succession of this grassland plant community. The stress-tolerance competition hypothesis suggests that tall plant species can obtain more light resources than short plant species (Tilman, Wedin & Knops, 1996). Therefore, due to competition for resources, the density of some species with low competitive abilities decreased or even disappeared after grazing exclusion (Bi et al., 2018). In the present study, grazing exclusion increased the abundance of *S. collina*, resulting in increased total aboveground biomass, indicating the tolerance of this species to grazing exclusion. However, their regeneration would have occupied more niche space in the community that thereby reduced the number of coexisting species possible (Wang et al., 2020). Therefore, grazing exclusion reduced the species richness, indicating a trade-off between the plant biomass and species richness in *S. glareosa* desert steppe after grazing exclusion (Bi et al., 2018).

We also found that grazing exclusion decreased the soil TOC. A previous study reported that perennial bunchgrasses were positively correlated with soil organic matter, total nitrogen, available nitrogen, and available potassium, implying that perennial bunchgrasses are an indicator of soil properties and can improve soil nutrients (Jing et al., 2014; Wang et al., 2020). Our study supported these observations. Thereby our results illustrate that eight-years of grazing exclusion on the *S. glareosa* desert steppe of the Inner Mongolia may not be fully conducive to the restoration of grassland ecosystem. These results are consistent with the previous studies on Tibetan Plateau, which show that eight years of grazing exclusion did not bring any ecological and economic benefits (Sun et al., 2020).

### Effects of grazing exclusion on soil microbial communities and interactions

Grazing exclusion had a positive effect on the bulk soil bacterial and fungal richness and diversity. These findings might be related to the high-productivity grassland without the disturbance of livestock (Zhang et al., 2018). Grazing impacts soil microbial communities by causing changes in plant composition and soil properties through trampling, defoliation, and urine deposition (Marcos, Bertiller & Olivera, 2019). Excluding livestock trampling increased plant coverage, enhanced soil aggregate structure, and therefore facilitate the soil microbial activity and growth (Wang et al., 2020). Thus, we interpret that the grazing exclusion provides more opportunities for different microbial species to interact with each other.

However, the soil bacterial and fungal networks responded differently to grazing exclusion, with fungal networks are more sensitive and quick response to grazing exclusion than that of bacterial networks. This may be because bacteria grow faster than fungi, while fungi may have more limited dispersal because of their larger size (Dassen et al., 2017). Fungal co-occurrence networks were characterized by high connectivity and low modularity, indicating low stability under grazing exclusion (Montoya, Pimm & Sole, 2006); while bacterial co-occurrence networks had weak interactions that suggest higher stability under grazing exclusion (Coyte, Schluter & Foster, 2015). We also found that changes in soil nutrient levels had positively associations with fungal networks. There is accumulating evidence that fungal communities were more responsive to sources of C and N than bacterial communities (Bardgett, Wardle & Yeates, 1998; Zhang et al., 2018). Grazing can promote root exudation and litter decomposition through defoliation and trampling, which can enhance the amount of C and N entering the soil (Bi et al., 2018). In contrast, grazing exclusion would be reduced the soil C and N pools in low fertility ecosystems, as suggested by previous researches (Bardgett, Wardle & Yeates, 1998; Sun et al., 2020). Therefore, grazing exclusion strengthened the soil fungal interactions by reducing the TOC, because more abundant members from fungal community were competing for limited niches (Lupatini et al., 2014). The more connected interactions and increased negative correlations in fungal networks confirmed these findings (Marcos, Bertiller & Olivera, 2019).

Not only were fungal network affected more by TOC, but fungal network also showed negative links to vegetation change. Fungal communities have been found to be the first consumers of belowground input of plant-derived C (De Vries et al., 2018). One recent study reported that mixed plant species were more likely than single-species to contain sufficient C, N, and P concentrations to satisfy the demand from microbial decomposers (Gong et al., 2020). This view is supported by our results. For example, when the fast-growing *S. collina* plant and fungal community compete for soil C and N—mainly arising from inputs of single-species litter—was insufficient to meet the growth rate of fungi (Wang et al., 2020). Conversely, in response to nutrient deficiency, fungal community could compete for limited nutrients, which are increased the fungal competition and thereby decreased their community stability (Marcos, Bertiller & Olivera, 2019; Wang et al., 2018). On the basis of the above reasoning, grazing exclusion may lead to a negative effect on soil fungal interactions and indicate an unstable and vulnerable fungal community in the *S. glareosa* desert steppe.

## Conclusions

Soil bacterial and fungal networks have different properties and respond differently to grazing exclusion, with fungal networks were more sensitive to grazing exclusion than bacterial networks. Eight years of grazing exclusion strengthened the soil fungal interactions by decreasing the plant richness and TOC, in this case triggering negative effects on the *S. glareosa* desert steppe. Our findings provide evidence that eight-years of grazing exclusion is not beneficial to plant diversity maintenance.

##  Supplemental Information

10.7717/peerj.9986/supp-1 Supplemental Information S1 Supplemental Figures and Tables

10.7717/peerj.9986/supp-2 Supplemental Information S2 Raw data: all plant, soil, and soil microbial variables

## Additional Information and Declarations

### DNA Deposition

The following information was supplied regarding the deposition of DNA sequences: The bacterial and fungal raw sequence data are available in the NCBI Sequence Read Archive: PRJNA627740 (SRP258186).

### Data Availability

The following information was supplied regarding data availability: The raw measurements are available in the Supplementary Files.
