# Supplementary material for: Soil fungal networks are more sensitive to grazing exclusion than bacterial networks
Source: PeerJ. 2020 Sep 18;8:e9986. doi: 10.7717/peerj.9986 (PMC7505065; doi:10.7717/peerj.9986)
Supplement: Supplemental Information S1 [file peerj-08-9986-s001.docx]

**Electronic Supplementary Material**

**Soil fungal networks are more sensitive to grazing exclusion than bacterial networks: a case study from a *Stipa glareosa* desert steppe of Inner Mongolia, China**

Lingling Chen, Jiajia Shi, Zhihua Bao, Taogetao Baoyin^*^^[[1]](#footnote-1)^

*Ministry of Education Key Laboratory of Ecology and Resource Use of the Mongolian Plateau & Inner Mongolia Key Laboratory of Grassland Ecology, School of Ecology and Environment, Inner Mongolia University,**Hohhot 010021, China*

**List of Supplementary Materials:**

**Figure S1** Overviews of the grazing and grazing exclusion study plots.

**Figure S2** Soil bacterial and fungal community composition of grazed and grazing excluded grassland soils based on the ten most abundant bacterial phyla and five most abundant fungal phyla.

**Figure S3** The correlations between module eigengenes and environmental factors in global networks of grazed and grazing excluded grassland soils.

**Table S1** ANOSIM test for differences in bacterial and fungal community composition in the rhizosphere and bulk soils of grazed and grazing excluded grassland soils

**Table S2** Topological properties of the empirical networks of bacteria and fungi communities in grazed and grazing excluded grassland soils.

**Table S3** Classification of nodes to identify putative bacterial keystone species in grazed and grazing excluded grassland soils.

**Table S4** Classification of nodes to identify putative fungal keystone species in grazed and grazing excluded grassland soils.

**Table S5** Spearman correlation analysis of plant characteristics, soil physicochemical properties, and microbial richness and diversity.


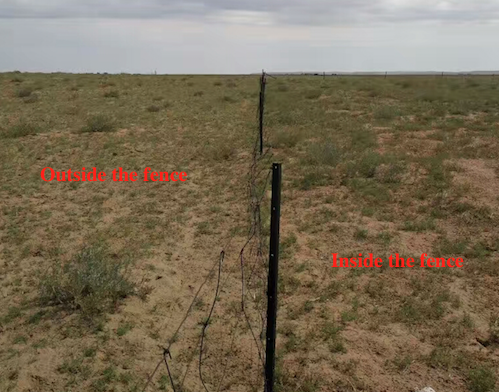


**Figure S1** Overviews of study sites.

**Figure S2** Soil bacterial (A) and fungal (B) community composition of grazed and grazing excluded grassland soils. GEr: rhizosphere soil of grazing excluded plot; GEb: bulk soil of grazing excluded plot; Gr: rhizosphere soil of grazed plot; and, Gd: bulk soil of grazed plots**
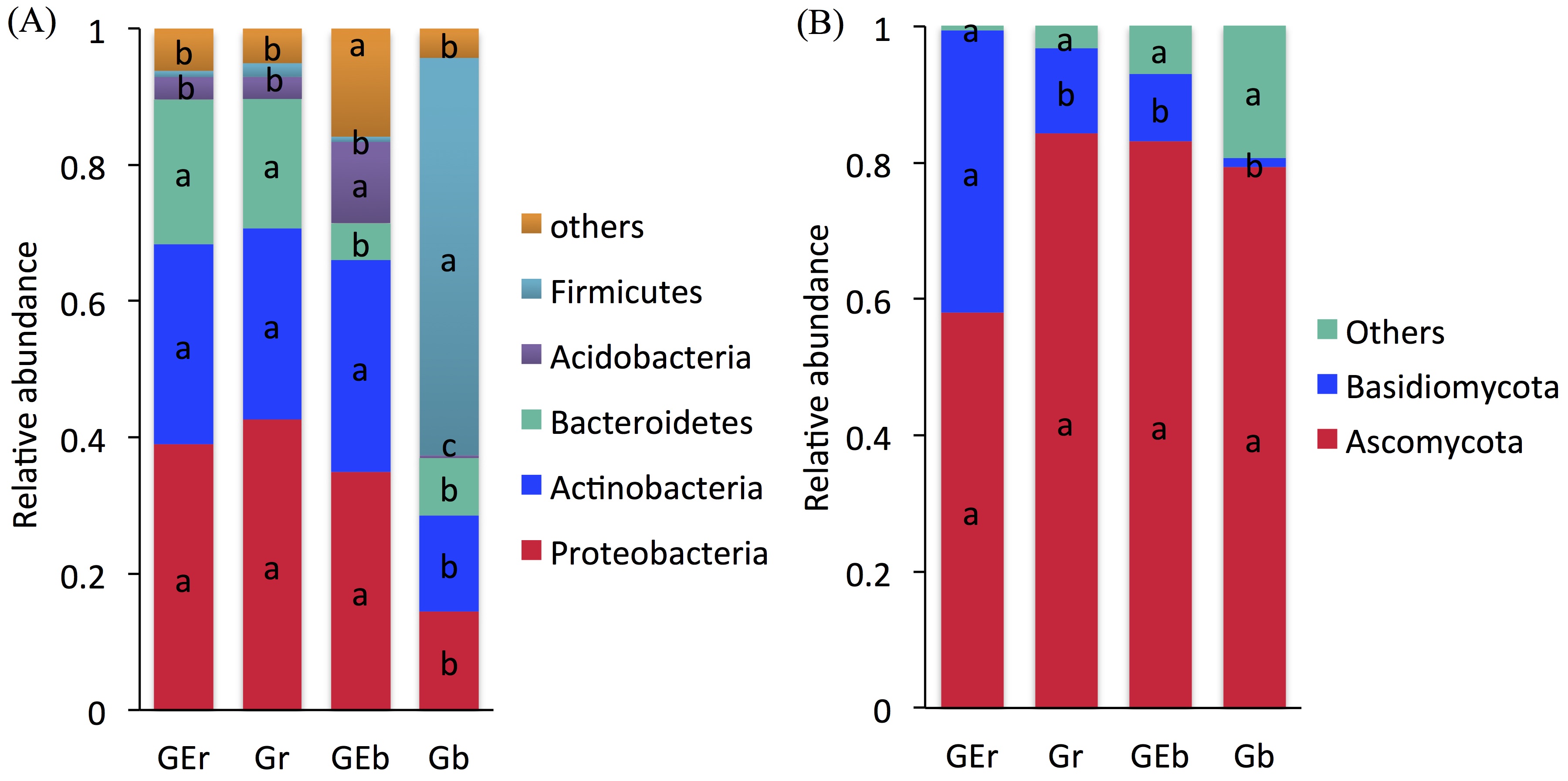
**.

**
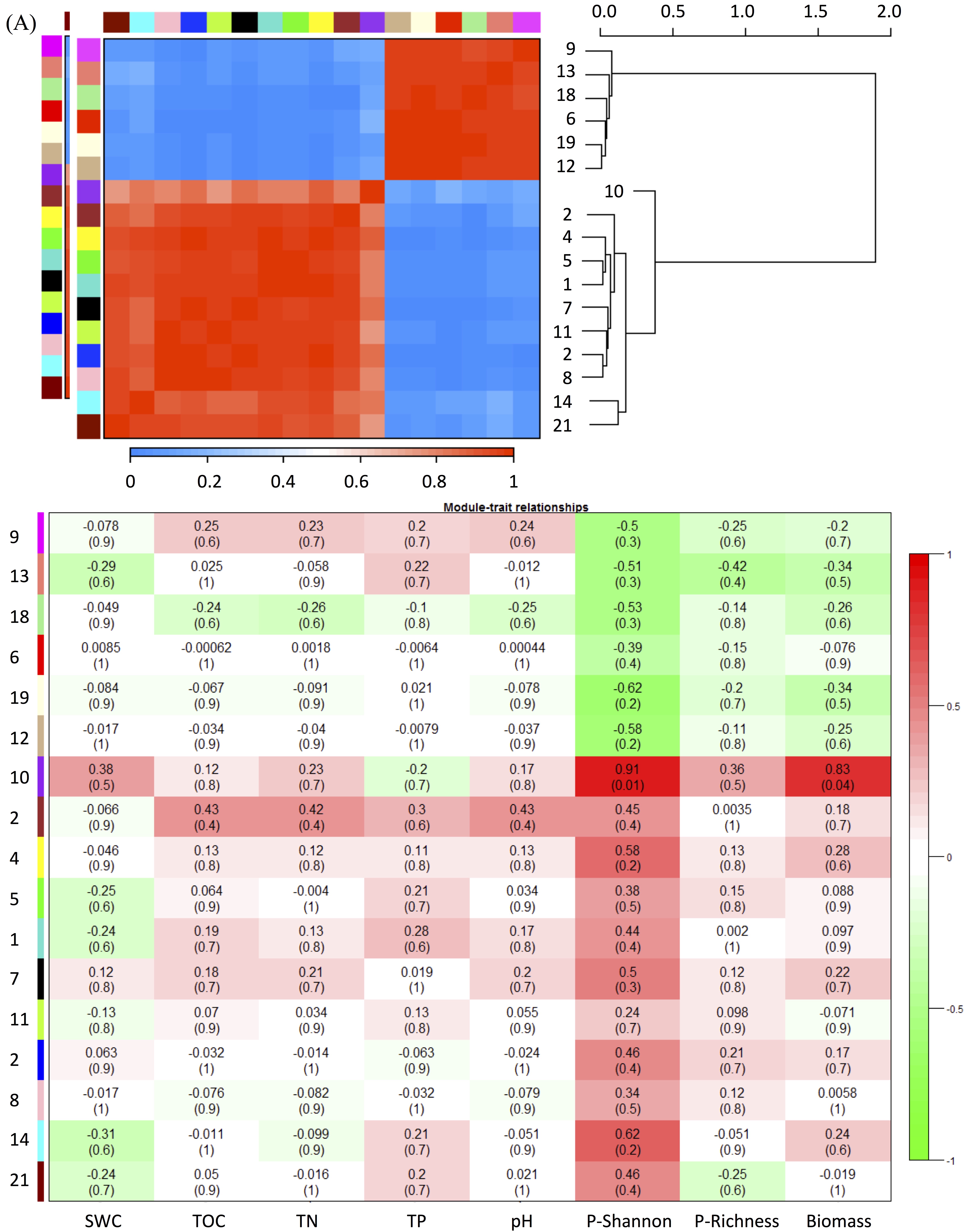
**

**
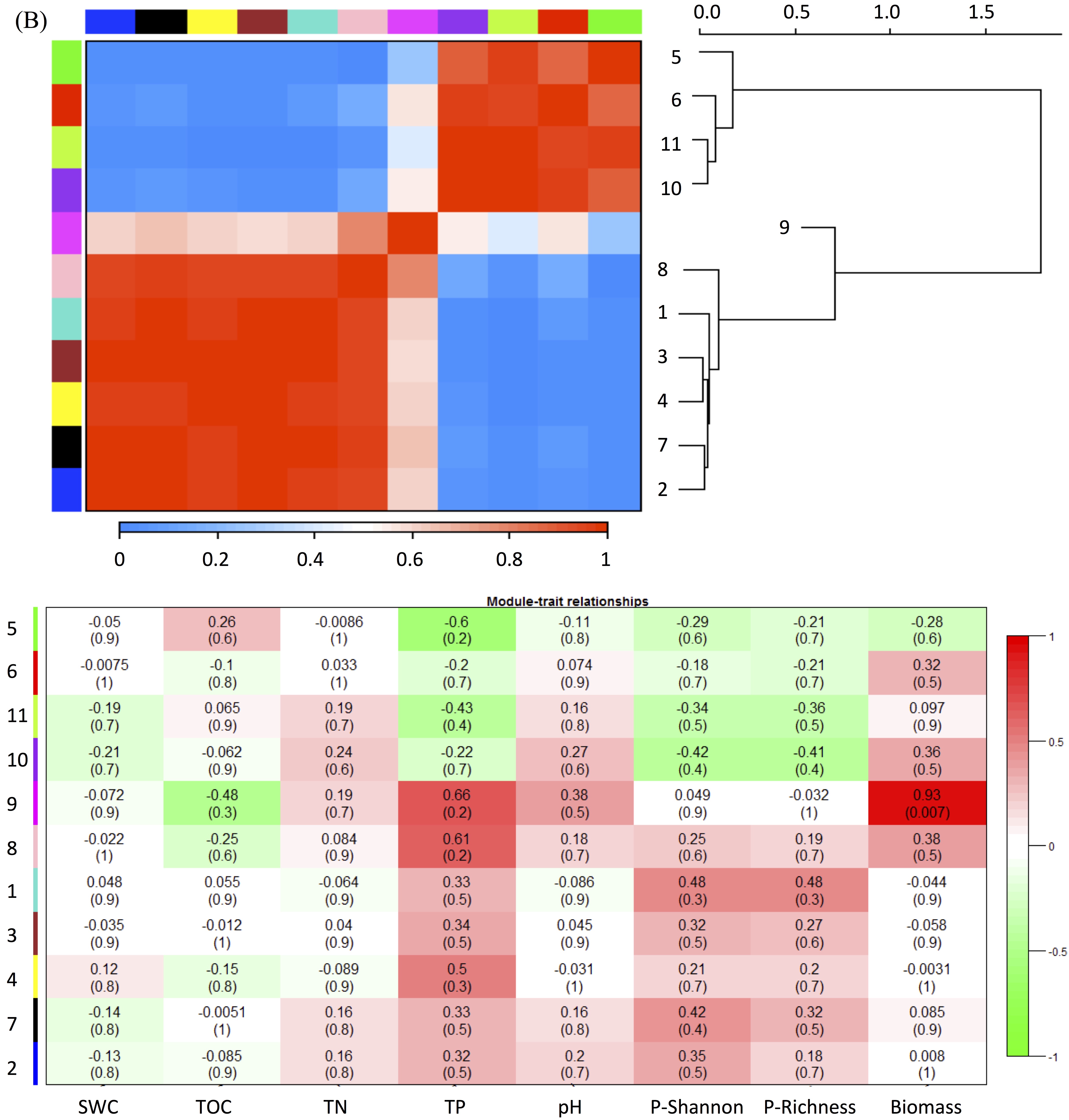
**

**
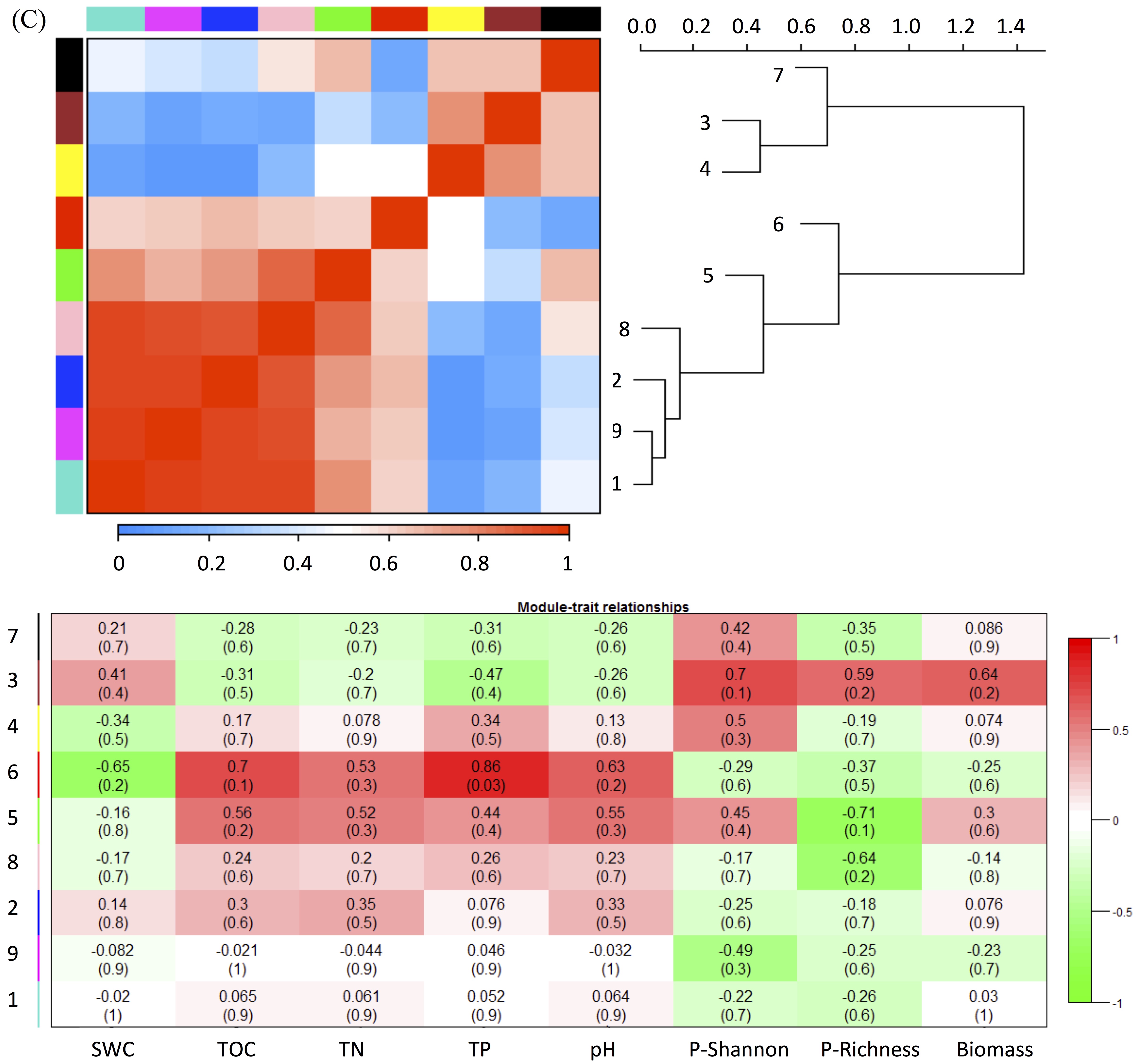
**

**
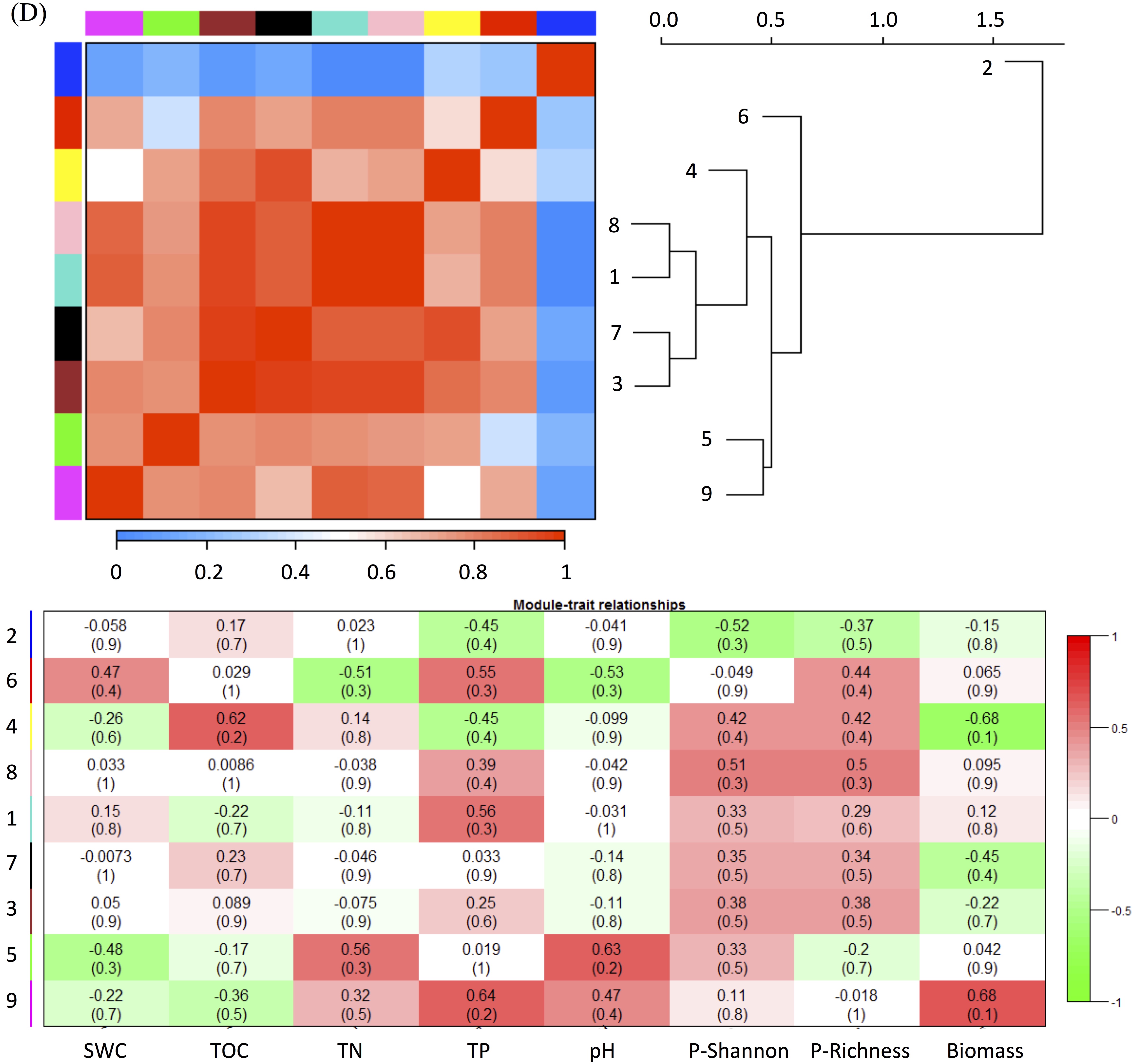
**

**Figure S3** The correlations between module eigengenes and environmental factors in global networks of grazed and grazing-excluded grassland soils. Red color of heatmap means highly positive correlation and green color means highly negative correlation. The numbers in each plot are the correlation coefficient (*r*) and significance (*p*) in parentheses. The environmental traits include soil water content (SWC), soil total organic carbon (TOC), soil total nitrogen (TN), soil total phosphorus (TP), soil pH value (pH), plant Shannon diversity, plant species richness and plant aboveground biomass. (A): Bacterial network of grazing-excluded grassland soil; (B): Bacterial network of grazed grassland soil; (C): Fungal network of grazing-excluded grassland soil; (D): Fungal network of grazed grassland soil.

**Table S1** ANOSIM test for differences in bacterial and fungal community composition in the rhizosphere and bulk soils of grazed and grazing excluded grassland soils

| **Group** | **16S** | |  | **ITS** | |
| --- | --- | --- | --- | --- | --- |
|  | ***R*** | ***P*** |  | ***R*** | ***P*** |
| GEr-Gr | 0.889 | 0.1 |  | 1 | 0.1 |
| GEb-Gb | 1 | 0.1 |  | 1 | 0.1 |
| GEr-GEb | 1 | 0.1 |  | 1 | 0.1 |
| Gr-Gb | 1 | 0.1 |  | 1 | 0.1 |

*R*-value is between (-1, 1), *R* > 0, indicates between groups differences. *R* < 0, indicates within-group difference are greater than between-groups, where differences were tested at *P* < 0.05. GEr: rhizosphere soil of grazing-excluded plot; GEb: bulk soil of grazing-excluded plot; Gr: rhizosphere soil of grazed plot; and, Gd: bulk soil of grazed plots.

**Table S2** Topological properties of the empirical networks of bacteria and fungi communities in grazed and grazing excluded grassland soils.

| **Topological characteristics** | | **16S** | |  | **ITS** | |
| --- | --- | --- | --- | --- | --- | --- |
|  |  | **G** | **GE** |  | **G** | **GE** |
| Commonly present OUT No. | |  |  |  |  |  |
| Empirical network | Total nodes | 697 | 869 |  | 262 | 355 |
|  | Total links | 4351 | 3589 |  | 701 | 1868 |
|  | Positive links | 3332 | 2452 |  | 486 | 1074 |
|  | Positive link percentage (%) | 76.58 | 68.32 |  | 69.33 | 57.50 |
|  | Average degree (avgK) | 12.485 | 8.260 |  | 5.351 | 10.524 |
|  | Average clustering coefficient (avgCC) | 0.438 | 0.368 |  | 0.414 | 0.399 |
|  | Harmonic geodesic distance (HD) | 4.141 | 5.047 |  | 4.482 | 3.607 |
|  | Average path distance (GD) | 5.366 | 6.759 |  | 6.073 | 4.763 |
|  | *R*^2^ of power-law | 0.818 | 0.850 |  | 0.709 | 0.731 |
|  | Similarity threshold | 0.972 | 0.985 |  | 0.960 | 0.950 |
|  | Modularity | 0.580 | 0.538 |  | 0.722 | 0.445 |
| Random networks* | Average clustering coefficient (avgCC) ±SD | 0.072±0.004 | 0.049±0.004 |  | 0.047±0.007 | 0.156±0.009 |
|  | Average harmonic geodesic distance (HD) ±SD | 2.747±0.011 | 3.096±0.015 |  | 3.048±0.025 | 2.599±0.019 |
|  | Average path distances (GD) | 2.989±0.017 | 3.352±0.023 |  | 3.388±0.038 | 2.858±0.028 |
|  | Average modularity±SD | 0.217±0.003 | 0.288±0.003 |  | 0.393±0.006 | 0.222±0.004 |

*Random networks were generated by rewiring all of the links of a corresponding empirical network with the identical nodes and links. Data were generated from 100 random runs and SD indicates the standard deviation from the 100 runs. G: Grazed area; GE: Grazing-excluded area.

**Table S3** Classification of nodes to identify putative bacterial keystone species in grazed and grazing excluded grassland soils

|  | **OUT No.** | **Phylum** | **Zi** | **Pi** | **Links** |
| --- | --- | --- | --- | --- | --- |
| **Grazing excluded area** | | | | | |
| **Connectors** | OTU_319 | *Acidobacteria* | -0.751 | 0.625 | 11 |
|  | OTU_616 | *Bacteroidetes* | -0.945 | 0.64 | 3 |
|  | OTU_1296 | *Proteobacteria* | -0.093 | 0.640 | 9 |
| **Module hubs** | OTU_25 | *Firmicutes* | 2.777 | 0 | 62 |
|  | OTU_36 | *Actinobacteria* | 2.515 | 0.064 | 59 |
|  | OTU_81 | *Bacteroidetes* | 2.657 | 0 | 22 |
|  | OTU_208 | *Bacteroidetes* | 5.021 | 0 | 22 |
|  | OTU_399 | *Bacteroidetes* | 2.973 | 0 | 15 |
|  | OTU_429 | *Proteobacteria* | 3.813 | 0 | 18 |
|  | OTU_469 | *Chloroflexi* | 3.367 | 0 | 19 |
|  | OTU_838 | *Acidobacteria* | *2.596* | *0* | *8* |
|  | OTU_1053 | *Actinobacteria* | *2.571* | *0* | *10* |
|  | OTU_1988 | *Gemmatimonadetes* | *2.709* | *0.346* | *9* |
|  | OTU_2481 | *Actinobacteria* | *2.874* | *0* | *17* |
|  | OTU_2483 | *Chloroflexi* | *3.234* | *0* | *8* |
| ***Grazed area*** | | | | | |
| **Connectors** | OTU_52 | *Acidobacteria* | -0.847 | 0.640 | 5 |
|  | OTU_189 | *Actinobacteria* | -0.824 | 0.625 | 8 |
|  | OTU_721 | *Bacteroidetes* | -0.874 | 0.625 | 12 |
|  | OTU_2553 | *Thermomicrobia* | -1.027 | 0.625 | 8 |
|  | OTU_3046 | *Proteobacteria* | -1.183 | 0.625 | 4 |
|  | OTU_3059 | *Actinobacteria* | -0.573 | 0.642 | 9 |
| **Module hubs** | OTU_66 | *Proteobacteria* | 2.700 | 0.278 | 54 |
|  | OTU_85 | *Bacteroidetes* | 2.537 | 0.236 | 22 |
|  | OTU_96 | *Proteobacteria* | 2.658 | 0.231 | 30 |
|  | OTU_144 | *Chloroflexi* | 3.387 | 0 | 53 |
|  | OTU_484 | *Chloroflexi* | 3.387 | 0 | 53 |
|  | OTU_1294 | *Proteobacteria* | 2.572 | 0 | 12 |

Modules hubs have *Zi* > 2.5 and *Pi* ≤ 0.62, whereas connectors have *Zi* ≤ 2.5 and *Pi* > 0.62.

**Table S4** Classification of nodes to identify putative fungal keystone species in grazed and grazing excluded grassland soils

|  | **OUT No.** | **Phylum** | **Zi** | **Pi** | **Links** |
| --- | --- | --- | --- | --- | --- |
| **Grazing excluded area** | | | | | |
| **Module hubs** | OTU_22 | *Ascomycota* | 3.152 | 0 | 9 |
|  | OTU_44 | *Ascomycota* | 2.544 | 0 | 14 |
|  | OTU_135 | *Glomeromycota* | 2.544 | 0 | 14 |
|  | OTU_172 | *Basidiomycota* | 2.544 | 0 | 14 |
| **Grazed area** | | | | | |
| **Connectors** | OTU_356 | *Basidiomycota* | -0.545 | 0.651 | 4 |
|  | OTU_499 | *Ascomycota* | -1.093 | 0.625 | 4 |
| **Module hubs** | OTU_144 | *Ascomycota* | 3.463 | 0.188 | 19 |
|  | OTU_396 | *Ascomycota* | 2.755 | 0.142 | 13 |
|  | OTU_508 | *Ascomycota* | 3.085 | 0.231 | 15 |

Modules hubs have *Zi* > 2.5 and *Pi* ≤ 0.62, whereas connectors have *Zi* ≤ 2.5 and *Pi* > 0.62

**Table S5** Spearman correlation analysis of plant characteristics, soil physicochemical properties, and microbial richness and diversity

| **Variable** | **PB** | **PR** | **PD** | **SWC** | **TOC** | **TN** | **TP** | **pH** | **BRR SWC** | **BDR TOC** | **BRB pH** | **BDB PD** | **FRR pH** | **FDR PD** | **FRB PR** | **FDB PB** |
| --- | --- | --- | --- | --- | --- | --- | --- | --- | --- | --- | --- | --- | --- | --- | --- | --- |
| PB | 1 |  |  |  |  |  |  |  |  |  |  |  |  |  |  |  |
| PR | -.883* | 1 |  |  |  |  |  |  |  |  |  |  |  |  |  |  |
| PD | -0.600 | 0.588 | 1 |  |  |  |  |  |  |  |  |  |  |  |  |  |
| SWC | .829* | -0.794 | -0.657 | 1 |  |  |  |  |  |  |  |  |  |  |  |  |
| TOC | -0.714 | 0.794 | .886* | -.886* | 1 |  |  |  |  |  |  |  |  |  |  |  |
| TN | -0.463 | 0.254 | .833* | -0.617 | 0.679 | 1 |  |  |  |  |  |  |  |  |  |  |
| TP | -0.169 | 0.348 | 0.169 | -0.507 | 0.507 | 0 | 1 |  |  |  |  |  |  |  |  |  |
| pH | -.812* | 0.716 | .870* | -.899* | .928** | 0.783 | 0.429 | 1 |  |  |  |  |  |  |  |  |
| BRR | 0.213 | -0.031 | 0.577 | -0.213 | 0.516 | 0.525 | 0.359 | 0.339 | 1 |  |  |  |  |  |  |  |
| BDR | -0.203 | 0.388 | .841* | -0.493 | .812* | 0.626 | 0.429 | 0.647 | .893* | 1 |  |  |  |  |  |  |
| BRB | -0.657 | 0.794 | .829* | -0.657 | .886* | 0.432 | 0.507 | .812* | 0.395 | 0.754 | 1 |  |  |  |  |  |
| BDB | -0.657 | 0.794 | .829* | -0.657 | .886* | 0.432 | 0.507 | .812* | 0.395 | 0.754 | 1.000** | 1 |  |  |  |  |
| FRR | -0.232 | 0.104 | 0.754 | -0.551 | 0.667 | .892* | 0.343 | 0.735 | 0.739 | 0.750 | 0.464 | 0.464 | 1 |  |  |  |
| FDR | -0.371 | 0.206 | 0.771 | -0.657 | 0.714 | .926** | 0.338 | .812* | 0.638 | 0.696 | 0.486 | 0.486 | .986** | 1 |  |  |
| FRB | .841* | -0.761 | -0.435 | .928** | -0.725 | -0.391 | -0.600 | -0.809 | 0.062 | -0.235 | -0.580 | -0.580 | -0.368 | -0.493 | 1 |  |
| FDB | .886* | -0.706 | -0.600 | 0.714 | -0.657 | -0.463 | -0.338 | -.841* | 0.152 | -0.232 | -0.714 | -0.714 | -0.377 | -0.486 | .812* | 1 |

The level of significance is as follows: ^**^0.001 < *P* <0.01, ^*^0.01 <*P* <0.05. PB: plant aboveground biomass; PR: plant species richness; PD: plant diversity; SWC: soil water content; TOC: soil total organic carbon; TN: soil total nitrogen; TP: soil total phosphorus; pH: soil pH value; BRR: bacterial richness in rhizosphere; BDR: bacterial diversity in rhizosphere; BRB: bacterial richness in bulk soil; BDB: bacterial diversity in bulk soil; FRR: fungal richness in rhizosphere; FDR: fungal diversity in rhizosphere; FRB: fungal richness in bulk soil; FDB: fungal diversity in bulk soil.

1. ^*^Corresponding author.

   E-mail address: E-mail: [bytgtnm@126.com](mailto:bytgtnm@126.com) (T. Baoyin) [↑](#footnote-ref-1)
